# Optimal DaTQUANT Thresholds for Diagnostic Accuracy of Dementia with Lewy Bodies (DLB) and Parkinson’s Disease (PD)

**DOI:** 10.3390/tomography10100119

**Published:** 2024-10-09

**Authors:** Phillip H. Kuo, Patrick Cella, Ying-Hui Chou, Alexander Arkhipenko, Julia M. Fisher

**Affiliations:** 1 Department of Radiology, City of Hope National Medical Center, Duarte, CA 91010, USA; phkuo@coh.org; 2 Departments of Medical Imaging, Medicine, and Biomedical Engineering, University of Arizona, Tucson, AZ 85724, USA; 3 GE HealthCare, Life Sciences, Imaging R&D, Marlborough, MA 01752, USA; patrick.cella@gehealthcare.com; 4 Department of Psychology, University of Arizona, Tucson, AZ 85721, USA; yinghuichou@arizona.edu; 5 GE HealthCare, Pharmaceutical Diagnostics, Marlborough, MA 01752, USA; alexander.arkhipenko@gehealthcare.com; 6 Statistics Consulting Laboratory, BIO5 Institute, University of Arizona, Tucson, AZ 85719, USA

**Keywords:** DaTQUANT, Parkinson disease, Parkinsonism, dementia with Lewy bodies, quantification, threshold, dopamine transporter, 123I-Ioflupane SPECT (123I-FP-CIT-SPECT), ioflupane, DaTscan

## Abstract

**Background:** Quantitative thresholds are helpful to define an abnormal DaT SPECT in patients with suspected nigrostriatal degenerative diseases (NSDD). The optimal DaTQUANT threshold for diagnostic accuracy of DaT SPECT across combined movement and cognitive disorder populations has been previously described. **Methods**: We established optimal DaTQUANT thresholds that enhance the discrimination between dementia with Lewy bodies (DLB) and non-DLB dementia types, as well as between Parkinsonian syndromes (PS) and conditions not characterized by nigrostriatal degeneration (non-PS). **Results:** Data from a total of 303 patients were used in this retrospective analysis. Posterior putamen of the more affected hemisphere (MAH) was shown to be an accurate single-variable predictor for both DLB and PS and was comparable to the most accurate multi-variable models. **Conclusions:** Automated quantification with DaTQUANT can accurately aid in the differentiation of DLB from non-DLB dementias and PS from non-PS. Optimal thresholds for assisting a diagnosis of DLB are striatal binding ratio (SBR) ≤ 0.65, z-score ≤ −2.36, and a percent deviation ≤ −0.54 for the posterior putamen of the MAH. Optimal posterior putamen thresholds for assisting a diagnosis of PS are SBR ≤ 0.92, z-score ≤ −1.53, and a percent deviation ≤ −0.33, which are similar to our previously reported posterior putamen threshold values using a blended patient pool from multiple study populations.

## 1. Introduction

Movement disorders such as Parkinson’s disease (PD) and related dementia disorders with alpha-synuclein pathology, such as multiple system atrophy and dementia with Lewy bodies (DLB), demonstrate loss of nigrostriatal dopaminergic neurons. In conjunction with patient medical history and clinical examination, evaluation of nigrostriatal dopamine transporters (DaT) in movement disorders can be performed with DaTscan™ (ioflupane iodine-123 [I^123^]) using single photon emission computed tomography (SPECT). DaT SPECT imaging evaluates the dopaminergic neuronal pathway via radiotracer uptake by presynaptic dopamine transporters in the striatum [1,2,3,4,5,6]. Detection of loss of DaT signal allows for differentiation between nigrostriatal degenerative disease (NSDD) and other non-NSDD entities, such as essential tremor (ET), vascular Parkinsonism, drug-induced Parkinsonism, or Alzheimer’s disease (AD), which do not involve degeneration of dopaminergic neurons, but may present with clinical features mimicking dopaminergic degeneration.

Semi-quantitative software has been developed to assist in the interpretation of DaT SPECT imaging using reproducible, standardized methods [7,8,9]. Semi-quantitative software can be optimized to be comparable to visual reads by highly experienced readers, potentially allowing inexperienced readers to increase their accuracy in everyday practice, as well as improving the confidence of experienced and inexperienced reader interpretations [10,11,12,13].

Despite the well-characterized algorithms for quantification of DaT SPECT imaging, scant guidance for clinical practice with approved software exists surrounding how to use the multitude of parameters for optimal diagnostic accuracy [14].

To date, there has been only limited published assessment of the most important quantitative parameter (or combination of parameters) and associated threshold values to distinguish normal from abnormal scans in clinical settings [15,16,17]. Meanwhile, in the research settings, quantitative thresholds vary significantly depending on the research question and patient population [12,18]. A z-score cut-off of −2 has been arbitrarily used by some for striatal and putaminal striatal binding ratio (SBR) to define an abnormal DaT SPECT in patients with suspected neurodegenerative Parkinsonism [19,20,21,22].

We previously evaluated DaTQUANT™ with ioflupane iodine-123 [I^123^] images acquired across multiple patient populations and imaging centers to develop guidelines for assisting with semi-quantitative interpretation of cases in routine practice [15]. Our earlier work recommended posterior putamen thresholds of SBR ≤ 1.0, a z-score of ≤−1.8, and percent deviation ≤ −0.34 [15], but these thresholds were calculated from a mixed dementia and movement disorder population which was primarily composed of patients with PS.

Following the approval of the use of DaTscan in patients suspected of having DLB by the FDA in 2022 [23,24], there has been an increased interest in clinical use of DaT SPECT as an indicative biomarker in patients with dementia. Therefore, separate quantitative thresholds for DLB and PS remain needed. It is important to note that DaT SPECT cannot reliably provide a differential diagnosis between these diseases, neither visually nor quantitatively.

Previously published work by Lanfranchi et al. aimed to define an optimal DaTQUANT z-score “cut-off” to differentiate DLB from AD and PD from ET, respectively [17]. Lanfranchi and colleagues found a posterior putamen z-score threshold of ≤−1.27 for optimal differentiation between PD and ET in patients with movement disorder, and a whole putamen z-score cut-off of ≤−0.96 to support diagnosis of DLB in patients with dementia.

Using a multicenter (MC) dataset from GE HealthCare clinical trials of DaTscan, we aimed to independently calculate optimal threshold values of quantitative variables for the individual populations of PS and DLB.

## 2. Materials and Methods

### 2.1. Study Population

This is an IRB-approved, retrospective review. The present analysis uses 303 patients, comprising the MC population from the three multicenter phase 3 or 4 clinical trials described previously by Neill et al. [15]. Two of the trials included 118 subjects in total: 78 with a clinical diagnosis of PS and 40 with non-PS [25,26]. One trial included 185 subjects: 73 with a clinical diagnosis of probable DLB (pDLB) and 112 with non-DLB forms of dementia, primarily AD [27]. Patients in these trials were evaluated for movement disorder (i.e., PS or non-PS) or dementia (due to DLB or other causes) and had a clinical diagnosis confirmed by an expert panel of clinicians at either one or three years of clinical follow-up.

### 2.2. Quantification

DaTQUANT™ v2.0 (GE HealthCare, Waukesha, WI, USA) is an FDA-approved, semi-quantitative software that enables the automated quantification of ioflupane iodine-123 [I^123^] images and comparison relative to normal population databases of ioflupane iodine-123 [I^123^] uptake.

The patients in the three multicenter clinical trials were imaged using gamma cameras having either two or three detectors fitted with low energy, high-resolution (LEHR) collimators. The images were acquired over a 30 min duration starting 3 to 3.5 h after injection of between 2.5 and 6 mCi (92–222 MBq) of ioflupane using an energy window of either 15% or 20% and a pixel size between 3 and 4.5 mm. Reconstruction was performed using the default DaTQUANT parameters of OSEM 2i10s and a 3D low-pass post-filter with cut-off frequency 0.6 cycles/pixel and power 10, and no corrections were applied. After automatic registration to the standard striatal template, the striatal and occipital volumes of interest were adjusted manually (only if necessary to accommodate any slight variations in patient anatomy).

Striatal binding ratios (SBR) were calculated using DaTQUANT for the striatum, putamen, caudate nucleus, anterior putamen, and posterior putamen bilaterally using three-dimensional volumes of interest. The formula used to calculate SBR was the difference in mean counts between the striatal region and background region (occipital cortex) divided by the mean counts in the background region. In addition, the putamen to caudate ratio (PCR) bilaterally and the caudate and putamen asymmetries (the ratio of left to right sides) were also calculated, resulting in a total of 14 variables from each exam for this analysis. For each variable, the number of standard deviations from the age-matched mean SBR of the normal database (z-score) and the percentage deviation from the age-matched mean value from the normal database (percent deviation) were also calculated.

### 2.3. Statistics

The analysis was performed in a manner similar to the previous work [15]. All analyses were conducted using R statistical language (v4.3.1; R Core Team 2021) [28]. For each participant and each of the 10 striatal regions of interest or PCRs, the most-affected hemisphere (MAH) was determined based on the lower z-score. The percent deviation and SBR from that hemisphere for that region of interest and participant were then used. Note that this definition of MAH could result in different hemispheres being selected for different striatal regions or PCRs in the same patient. The SBR, percent deviation, and z-score measurements for the six MAH, along with the putamen and caudate asymmetries, were then used in a series of logistic regression models to predict disease state.

For each ioflupane iodine-123 [I^123^] binding measure (i.e., SBR, z-score, percent deviation), every possible combination of one to eight measurements (255 combinations in total) was included in a separate logistic regression model of disease state. For example, the most complex model using z-scores modeled the log odds of disease as a linear combination of the MAH z-scores for the striatum, putamen, caudate nucleus, anterior putamen, posterior putamen, and PCR, and the z-scores for the caudate and putamen asymmetries. In contrast, the simplest models included only a single predictor (e.g., the MAH z-score for the putamen). The predictive ability (accuracy, sensitivity, and specificity) of each combination of measurements and ioflupane iodine-123 [I^123^] binding measure was evaluated using leave-one-out cross-validation. Specifically, the disease state of each left-out data point was predicted using the model fit to the remaining data and a set of 21 equally spaced thresholds from 0 to 1. If the estimated disease probability of the test point was at or above a given threshold, the patient was predicted to have pDLB or PS. Accuracy, sensitivity, and specificity were calculated for each combination of model and threshold from the results for the left-out data.

Results were determined from the best-performing multi-predictor models and from all single-predictor models for each ioflupane iodine-123 [I^123^] binding measure. Two-sided bootstrap case cross-validation 95% confidence intervals on accuracy were calculated from 1000 bootstrap datasets. These confidence intervals were calculated using a slight modification of the one-sided intervals in Jiang et al. [29]. Model equations were estimated for the best models by refitting the optimal combinations of predictors to the full dataset. Additionally, the SBR, percent deviation, and z-score thresholds associated with the predictive switch from non-DLB to pDLB and from non-PS to PS were provided for the single-predictor models.

Previously described thresholds from Neill et al. [15] and Lanfranchi et al. [17] of the posterior putamen or whole putamen were applied to our data. The corresponding performance (accuracy, sensitivity, specificity) on our data was assessed. However, it should be noted that the thresholds developed by Lanfranchi and colleagues were based on a different patient population, and only z-score thresholds were published. Additionally, the thresholds from Neill et al. [15] were developed using a dataset that contained the data used in these analyses. Thus, the Neill et al. [15] thresholds are not independent of the current data.

## 3. Results

### 3.1. Demographics

The main characteristics of our MC patient population are summarized in Table 1. The mean age of participants was higher for dementia (74 years) than for movement disorder (66 years), with patient ages ranging from 54 to 89 years in the dementia group compared to 37 to 87 years in the movement disorder group. The proportion of males to females was similar between the two groups.

### 3.2. Putamen Distributions, Optimal Variables, and Thresholds for Dementia

**Figure 1:**
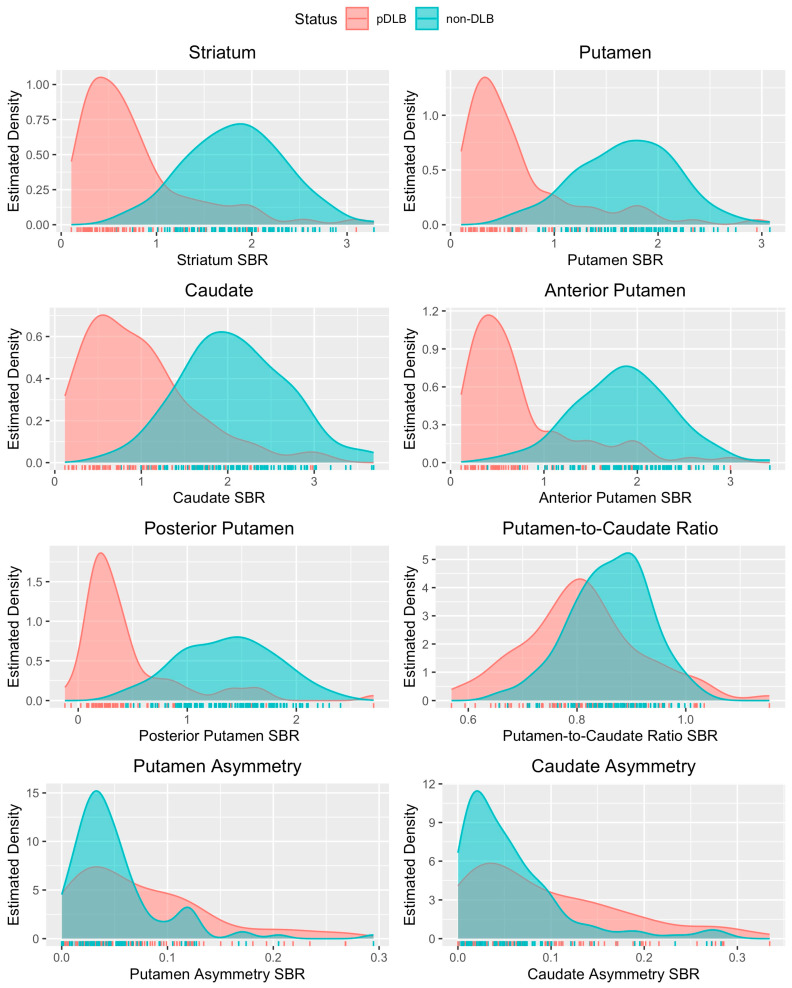
Estimated distributions of the SBR for each MAH striatal sub-region in patients evaluated for dementia.

Density plots were created for the estimated distributions of the SBR, z-score, and percent deviation. As an example, the density plots for the estimated distributions of the SBRs in patients with dementia are shown in Figure 1 and shows that almost all the non-asymmetry neurological variables were predictive of disease state, with the posterior putamen showing the clearest distinction.

The most accurate multi-variable and single-variable models for prediction of pDLB are presented in Table 2. We selected models with the highest accuracy and only considered models with a specificity greater than or equal to 0.90. In single- versus multi-variable analysis, the posterior putamen of the MAH demonstrated the highest accuracy, with sensitivity above 0.80. The differences in accuracy, sensitivity, and specificity between single-variable models using the posterior putamen and the best-performing multi-variable models ranged from 0.00 to 0.05. Optimal posterior putamen thresholds for assisting a diagnosis of DLB are SBR ≤ 0.65, z-score ≤ −2.36, and a percent deviation ≤ −0.54. The estimates of accuracy, sensitivity, and specificity for these thresholds and for example best-performing multi-variable models in patients presenting with dementia are shown in Table 2.

**Figure 2:**
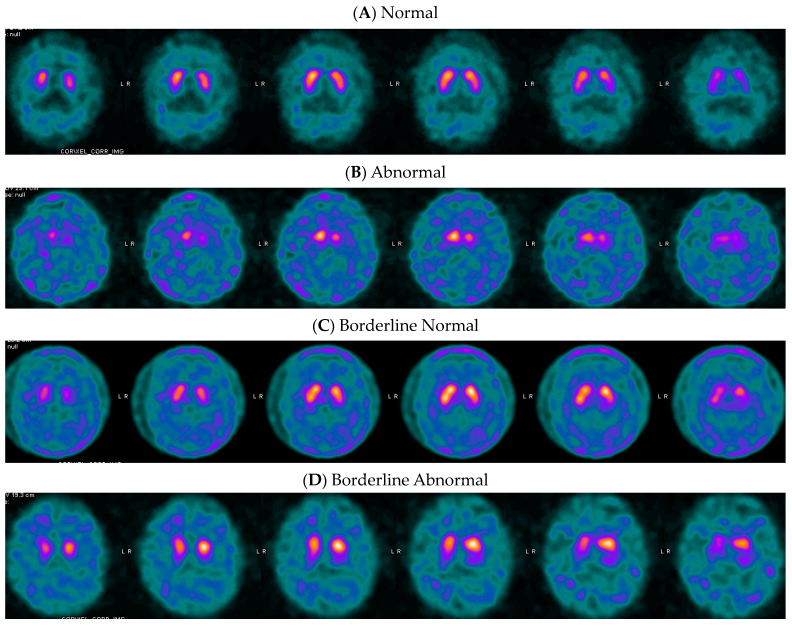
Examples of ioflupane iodine-123 [I^123^] images from patients with primary symptoms of dementia. (**A**) Negative: a 77-year-old man’s DaT imaging resulted in an MAH posterior putamen SBR of 1.00, z-score of −1.17, and a −28% deviation. These quantification values were greater than the optimal thresholds and, as expected, were in agreement with the final clinical diagnosis of non-DLB. (**B**) Positive: a 54-year-old man’s DaT imaging resulted in a MAH posterior putamen SBR of 0.15, z-score of −4.79, and a −92% deviation, and agreed with the final clinical diagnosis of pDLB. (**C**) Borderline negative: a 66-year-old man’s DaT imaging resulted in an MAH posterior putamen SBR of 0.73, z-score of −2.86, and a −52% deviation. In this borderline case, only the SBR and percent deviation aligned with the clinical diagnosis of non-DLB. (**D**) Borderline positive: an 83-year-old woman’s DaT imaging resulted in a MAH posterior putamen SBR of 0.39, z-score of −2.97, and a −71% deviation, and agreed with the final clinical diagnosis of pDLB.

Figure 2 provides examples of DaT SPECT images in dementia patients with MAH posterior putamen SBR values above, below, and close to the threshold value. It is worth noting that images with quantified values near the thresholds are often difficult to visually classify as normal or abnormal.

### 3.3. Optimal Variable and Threshold for Movement Disorders

The best multi-variable and single-variable (posterior putamen) models for movement disorders are presented in Table 3. As with dementia patients, we only considered models with a specificity greater than or equal to 0.90. Unlike in the dementia group, the multi-variable models demonstrated slightly higher accuracy than the single-variable models. However, the accuracy of the MAH posterior putamen was only lower by 0.01 to 0.02. All selected multi-variable models with specificity equal to or above 0.90 and accuracy equal to or above 0.80 maintained a sensitivity above 0.70, with the models shown in Table 3 having sensitivities notably above that. Overall, there were no substantial differences in sensitivity or specificity between the best-performing multi-variable models versus the single posterior putamen, with the differences in sensitivity or specificity ranging from 0.00 to 0.03. Optimal MAH posterior putamen thresholds for assisting a diagnosis of PS are SBR ≤ 0.92, z-score ≤ −1.53, and percent deviation ≤−0.33. Accuracy, sensitivity, and specificity at these thresholds in comparison with either the best-performing multi-variable model or an example of one of the best-performing multi-variable models are shown in Table 3.

**Figure 3:**
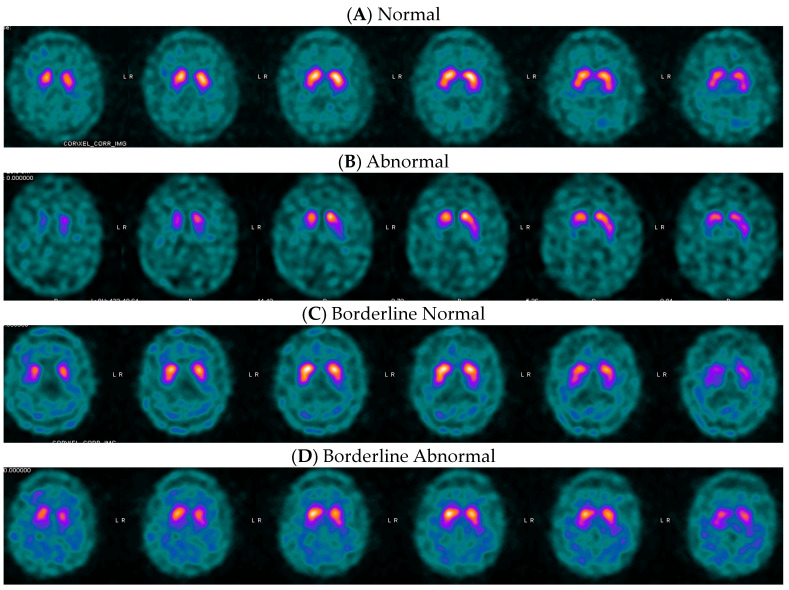
Examples of ioflupane iodine-123 [I^123^] images from patients with primary symptoms of movement disorder. (**A**) Negative: a 60-year-old man’s DaT imaging resulted in a MAH posterior putamen SBR of 1.42, z-score of −0.65, and a −14% deviation, which were above the thresholds and agreed with the final clinical diagnosis of non-PS. (**B**) Positive: an 83-year-old woman’s DaT imaging resulted in a MAH posterior putamen SBR of 0.86, z-score of −1.53, and a −37% deviation. In this case, the quantified values were close to the thresholds due to a photopenic area in the occipital region which artifactually lowered the activity in the background region of interest, but the images are clearly visually abnormal due to putamen differences between the left and right hemispheres. Final clinical diagnosis was PS. (**C**) Borderline Negative: an 84-year-old man’s DaT imaging resulted in a MAH posterior putamen SBR of 0.96, z-score of −0.36, and a −28% deviation, which agreed with the final clinical diagnosis of non-PS. (**D**) Borderline Positive: a 63-year-old man’s DaT imaging resulted in a MAH posterior putamen SBR of 0.83, z-score of −2.11, and a −48% deviation, which agreed with the final clinical diagnosis of PS.

Examples of DaT SPECT images of movement disorder patients with MAH posterior putamen SBR above, below, and close to the threshold values are provided in Figure 3. Please note the difficulty of visual binary interpretation of cases C and D. In these cases, quantification may increase confidence in scan interpretation, as previously described by Booij and colleagues [10].

### 3.4. Comparison with Previously Published Thresholds

When our previous threshold values for SBR, z-score, and percent deviation (calculated using three different populations, including the MC dataset) were applied to the MC dataset alone, the results were better for the movement disorder population than the dementia population. As seen in Table 4, the accuracy, sensitivity and specificity in the movement disorder population are similar to both the previously published values and the single-variable models in the present study. In the dementia population, sensitivity is higher, and specificity is lower, due to the previous threshold values being higher than the optimal threshold value for the same region calculated for the dementia population alone in the present study.

The performance of the Lanfranchi et al. [17] threshold values when applied to our MC dataset can be seen in Table 4, showing a similar accuracy, sensitivity, and specificity for our movement disorder patients compared to results using both our previously published threshold for z-score and the best performing single-variable model in the present study. In the dementia population from the MC dataset, accuracy and specificity were considerably lower than when using our previously published threshold z-score or the best-performing single-variable model in the present study. Sensitivity was higher (88%) due to the higher threshold value for the whole putamen z-score used by Lanfranchi et al. [17]

## 4. Discussion

### 4.1. Posterior Putamen as Optimal Single-Variable Model

The posterior putamen region in DaTQUANT allows for a robust differentiation of diseases with and without nigrostriatal degeneration, albeit using different optimum threshold values for DLB and PS. We propose using the single-variable posterior putamen predictor versus multi-variable prediction models. Multi-variable predictors theoretically can provide a more holistic view and potentially increase accuracy by simultaneously accounting for the more complex patterns of dopamine transporter loss across different brain regions. This may also reduce the risk of omitted-variable bias, which can occur when important predictors are not included in the analysis. An optimal discriminator will balance simplicity of use, ease of interpretability and adequate diagnostic performance. A single-variable predictor, such as the z-score of the MAH posterior putamen, is much simpler to use and interpret than a weighted multi-variable calculation. Indeed, our work has shown that overall, there were no substantial differences in accuracy, sensitivity, or specificity between the best-performing multi-variable models and the single posterior putamen variable. Moreover, the critical importance of the posterior putamen as a single variable correlates with the expected natural history of the disease processes for both DLB [27,30] and PS [31]. Thus, the use of the single-variable model based on the MAH posterior putamen is recommended because it is simple to analyze and interpret.

### 4.2. Comparison with Previously Described Clinical DaTQUANT Thresholds

In our dataset, we have found that optimal thresholds for assisting a differential diagnosis of probable DLB vs non-DLB are SBR ≤ 0.65, z-score ≤ −2.36, or percent deviation ≤ −0.54 of the posterior putamen in the MAH. Optimal posterior putamen threshold values for the MAH to assist a differential diagnosis of PS vs non-PS are SBR ≤ 0.92, z-score ≤ −1.53, or a percent deviation ≤ −0.33. Comparative performance in our dataset of earlier-published thresholds is shown in Table 4.

The separate DLB and PS threshold values are similar to those previously calculated over three study populations pulled together by Neill et al. [15]. In our previous publication, a multi-variable and single-variable evaluation of optimal quantitative variables and thresholds in DaTQUANT™ showed that the posterior putamen as a single variable consistently yielded a high accuracy in diagnosis across the three different study populations. The current expansion of that work split the multicenter (MC) population from that publication into dementia and movement disorder populations, acquired close to real-world conditions and across multiple centers. Separate predictive models and thresholds were evaluated for dementia and movement disorder patients to determine if there was a difference.

Our thresholds for DLB were more accurate than the thresholds for PS, although there was an overlap in confidence intervals. Analysis of movement disorder patients separately from dementia patients yielded more permissive thresholds, which were closer to the cut-off z-score values found by Lanfranchi and colleagues.

Based on Lanfranchi and colleagues’ findings, as well on the observations that DLB sometimes presents with fairly symmetric (anterior-posterior and/or right-left) generalized loss of DaT binding [23], we expected whole striatal signal or multi-variable models to be more predictive of DLB. Surprisingly, and in contrast to the findings of Lanfranchi and colleagues, we have found that MAH posterior putamen more accurately discriminates pDLB from non-DLB in our MC dataset.

It is difficult to directly compare our findings to the work of Lanfranchi and colleagues. It should be noted that their work was a single-center study based on a different patient population. Our work does not specifically address DLB vs AD, but pDLB vs non-DLB dementia. In addition, patients who were diagnosed in the recruiting study with possible DLB (high uncertainty as to whether they truly had DLB) were not included in the MC dataset. Thus, it is not possible to directly compare accuracies between the two studies.

### 4.3. Clinical Implication of Differential Thresholds for DaT SPECT

DaT SPECT does not have the ability to reliably differentiate between PS and pDLB. It is used to differentiate conditions with nigrostriatal degeneration from more benign conditions with a largely preserved nigrostriatal system. However, differential thresholds allow the optimal use of quantification to aid visual DaT SPECT interpretation in distinct clinical scenarios of either dementia or movement disorder.

In routine clinical use, the absence of disease-modifying treatments for Parkinsonian syndromes justifies the acute need to avoid false positive diagnosis of diseases with nigrostriatal loss. In the present study, we deliberately limited our models to those with high specificity (equal or superior to 0.90).

Distinguishing patients with pDLB from other dementias without dopaminergic neurodegeneration can be challenging, even after repeat observation. Indeed, Walker reported on a series of 20 dementia patients with and without Lewy bodies in whom there was no significant difference at baseline between the neuropathologically diagnosed DLB and non-DLB groups with respect to any of the demographic or clinical characteristics, including the frequency of Parkinsonian signs, visual hallucinations, and clinical fluctuation [30]. Use of biomarkers, and specifically DaT SPECT, is improving the accuracy of clinical diagnosis in DLB [27,32], but interpretation of the DaT SPECT in dementia presents its own challenges [23].

DaT SPECT quantification is a helpful tool to increase the accuracy and confidence of visual interpretation, especially for inexperienced readers [10]. We propose using optimized quantitative thresholds to aid differential diagnosis of PS from non-PS and dementia with and without Lewy body pathology following imaging with ioflupane iodine-123 [I^123^]. Although in both clinical scenarios, we recommend using values from the posterior putamen, threshold values for DLB are substantially more restrictive than those for PS. This could be in part explained by the more advanced age of the patient population with dementia, or the fact that all the patients with dementia who were recruited in the study had progressed beyond the mild cognitive impairment (MCI) phase of symptoms by the time of ioflupane iodine-123 [I^123^] imaging. Patients with dementia symptoms typically have more comorbidities in the brain, which may lower the DaT density in the striata slightly and result in more restrictive optimum threshold values to differentiate pDLB from non-DLB pathology (see Figure 2C for an example). We can also speculate that the pathophysiology of PS and DLB could possibly explain differences in nigrostriatal pathway alterations [22].

While these optimized thresholds for quantitative differentiation may help with clarity and confidence in reporting, they should not replace visual assessment of images or be used as absolute cut-off values. Patient quantitative values that are closer to the thresholds hold more uncertainty in the prediction than those further from the threshold values. Binary classification of DaT SPECT scans presents a challenge, as clinicians are expected to give binary interpretation to the continuous biological process of the loss of dopaminergic synapses [33]. Borderline scans are challenging for visual assessment and might remain indeterminate after quantitative analysis. As shown in Figure 2 (panel C) and Figure 3 (panel B), uncertain, difficult-to-interpret cases sometimes correspond to borderline quantification values that cannot be accurately classified as normal or abnormal. These scans can remain inconclusive, even after quantification.

Additionally, the clinical interpretation of the normal DaT SPECT in DLB should be taken with caution, as several earlier publications suggested that a negative DaT SPECT can be observed in approximately 10% of DLB patients at the onset of Parkinsonian symptoms [34]. The current work did not address the differentiation of movement disorder and dementia diagnoses based on DaT SPECT images or their quantification alone.

### 4.4. Research Context, Future Perspectives, and Study Limitations

The use of molecular imaging in the diagnosis of patients presenting with movement disorders and dementia is evolving. As mentioned earlier, detection of DLB has become an approved indication for ioflupane iodine-123 [I^123^] in the United States [23,24], and further work is needed to refine the interpretation of DaT SPECT in dementia patients. Our work, together with Lanfranchi et al. [17], addresses the need for non-arbitrary, evidence-based DaT-SPECT quantitative thresholds to aid in differentiation of NSDD, not from healthy volunteers but from patients without NSDD assessed by DaT SPECT in a distinct clinical scenario of dementia.

Additional analysis of quantification of DaT SPECT in the diagnosis of DLB is being conducted by the US Dementia with Lewy Bodies Consortium [35], and their results remain to be published. Overall, there is a need for harmonization of DaTQUANT thresholds developed independently on different datasets. It is possible that further work will unify thresholds by establishing a “gray zone” that will delineate the value ranges of indeterminate scans.

Future directions might include the evaluation of thresholds in other evolving clinical scenarios such as prodromal patients, those with rapid eye movement sleep behavior disorder (RBD), or patients who have tested positive on new synuclein tests [36]. Biomarker-based staging systems for synucleinopathies are now debated by the research community, indicating that there is a growing need for precise and standard DaT SPECT thresholds [37]. Current work will benefit from harmonization efforts across software platforms, and possibly even across tracers.

There are limitations to the current study. It applies only to ioflupane iodine-123 [I^123^] with DaTQUANT-calculated values. In addition, the accuracy of DaTQUANT relies heavily on the quality of the SPECT imaging, and poor imaging can lead to inaccurate quantification and misdiagnosis [16]. Anatomic abnormalities affecting the striatal or background occipital regions can also result in errors. Another limitation is the clinical diagnosis of each patient. Clinical diagnosis in this study was performed between 2002 and 2007 with the diagnostic criteria available at the time. For the dementia patients, only the information available in the medical records for each patient was used by an expert panel to make the final clinical diagnosis (i.e., there was no patient visit with the panel or any of its members). Our work with dementia patients needs to be expanded to use larger cohorts, ideally with autopsy-validated diagnosis or a combination of clinical diagnosis with emerging biomarkers.

## 5. Conclusions

In summary, we have found that optimal thresholds for assisting a diagnosis of probable DLB are SBR ≤ 0.65, z-score ≤ −2.36, or a percent deviation ≤ −0.54 of the posterior putamen in the MAH. Optimal posterior putamen threshold values for the MAH to assist a differential diagnosis of movement disorder with NSDD are SBR ≤ 0.92, z-score ≤ −1.53, or a percent deviation ≤ −0.33, as summarized in Table 5. These thresholds should complement and not replace the visual assessment of DaT SPECT. Scans with ambiguous visual interpretation and quantitative values close to the threshold might remain indeterminate.

## Figures and Tables

**Table 1:** Baseline Demographics.

	Dementia	Movement Disorder	Total
Total	185	118	303
Sex			
Male	102 (55%)	67 (57%)	169 (56%)
Female	83 (45%)	51 (43%)	134 (44%)
Age *	73.78 (±7.2)	66.34 (±10.99)	70.88 (±9.57)
NSDD **	73 (39%)	78 (66%)	151 (49%)

* Mean (±standard deviation); ** Nigrostriatal degenerative diseases (NSDD) are represented by probable DLB for dementias and Parkinsonian syndromes for movement disorder populations.

**Table 2:** Estimates for best-performing models in patients with dementia.

		Single-Variable Model Posterior Putamen	Example of Best-Performing Multi-Variable Model
SBR			Post Put + PCR
	Accuracy	0.90 [0.85, 0.94]	0.89 [0.84, 0.94]
	Sensitivity	0.81	0.80
	Specificity	0.96	0.96
	Threshold	0.65	Not applicable
z-score			Striatum + Caudate + PCR
	Accuracy	0.89 [0.84, 0.94]	0.89 [0.83, 0.94]
	Sensitivity	0.80	0.75
	Specificity	0.96	0.97
	Threshold	−2.36	Not applicable
% Dev			Post Put + PCR
	Accuracy	0.90 [0.85, 0.94]	0.89 [0.84, 0.94]
	Sensitivity	0.81	0.80
	Specificity	0.96	0.96
	Threshold	−0.54	Not applicable

[95% confidence intervals].

**Table 3:** Estimates for best-performing Models in patients with movement disorders.

		Single-Variable Model: Posterior Putamen	Example of Best Performing Multi-Variable Model
SBR			Striatum + Caudate
	Acc	0.82 [0.75, 0.89]	0.83 [0.75, 0.90]
	Sens	0.77	0.78
	Spec	0.93	0.93
	Threshold	0.92	Not applicable
z-score			Striatum + Post Put + Caud Asy
	Acc	0.83 [0.76, 0.90]	0.84 [0.75, 0.92]
	Sens	0.78	0.80
	Spec	0.93	0.93
	Threshold	−1.53	Not applicable
% Dev			Striatum + Post Put + Caud Asy
	Acc	0.82 [0.75, 0.89]	0.84 [0.74, 0.92]
	Sens	0.78	0.80
	Spec	0.90	0.93
	Threshold	−0.33	Not applicable

[95% confidence intervals].

**Table 4:** Performance of the Neill et al. [15] and Lanfranchi et al. [17] thresholds when applied to the patients in the MC dataset.

		Neill et al. 2021 [15]		Lanfranchi et al. 2023 [17] ^a^	
		Post Put—Movement Disorders	Post Put—Dementia	Post Put—Movement Disorders	Put—Dementia
SBR	Acc	0.82	0.82	-	-
	Sens	0.78	0.89	-	-
	Spec	0.90	0.78	-	-
	Threshold	1.0	1.0	-	-
z-score	Acc	0.81	0.87	0.82	0.81
	Sens	0.74	0.85	0.78	0.88
	Spec	0.92	0.88	0.90	0.77
	Threshold	−1.8	−1.8	−1.27	−0.96 **
% Dev	Acc	0.82	0.86	-	-
	Sens	0.78	0.89	-	-
	Spec	0.90	0.84	-	-
	Threshold	−0.34	−0.34	-	-

^a^ Lanfranchi et al. [17] only reported the performance of z-score threshold values. ** Threshold values for whole putamen reported in Lanfranchi et al. [17] cannot be directly compared to posterior putamen threshold values.

**Table 5:** Summary of differential thresholds for dementia and movement disorders.

	Posterior Putamen	
	Dementia	Movement Disorders
SBR Threshold	0.65	0.92
z-score Threshold	−2.36	−1.53
Percent Dev Threshold	−0.54	−0.33

Thresholds calculated in our study ensure accuracy ranging from 0.84 to 0.94 for dementia and 0.75 to 0.90 for movement disorders. Please see Table 2 and Table 3 for detailed sensitivity and specificity.

## Data Availability

The datasets presented in this article are not readily available because they are the property of GE HealthCare as part of company-sponsored clinical trials used in regulatory submissions. The datasets were made available to the authors under a research agreement as part of an investigator sponsored trial. Requests to access the datasets should be made via the investigator-sponsored trial process at GE HealthCare and directed to medical.affairs@gehealthcare.com.
